# Incidence of low-grade infection in aseptic loosening of total hip arthroplasty: A prospective multicenter study using extensive routine and broad-range 16S PCR with reverse line blot diagnostics

**DOI:** 10.3109/17453674.2010.525201

**Published:** 2010-11-26

**Authors:** Dirk Jan F Moojen, Gijs van Hellemondt, H Charles Vogely, Bart J Burger, Geert H I M Walenkamp, Niek J A Tulp, B Wim Schreurs, Frank R A J de Meulemeester, Corrie S Schot, Ingrid van de Pol, Takaaki Fujishiro, Leo M Schouls, Thomas W Bauer, Wouter J A Dhert

**Affiliations:** ^1^Department of Orthopaedics, University Medical Center Utrecht, Utrecht, the Netherlands; ^2^Department of Orthopaedics, Sint Maartensclinic, Nijmegen, the Netherlands; ^3^Department of Orthopaedics, Medical Center Alkmaar, Alkmaar, the Netherlands; ^4^Department of Orthopaedics, Maastricht University Medical Center, Research Institute CAPHRI, Maastricht, the Netherlands; ^5^Department of Orthopaedics, Isala Clinics, Zwolle, the Netherlands; ^6^Department of Orthopaedics, University Medical Center Sint Radboud, Nijmegen, the Netherlands; ^7^Department of Orthopaedics, Onze Lieve Vrouwe Gasthuis, Amsterdam, the Netherlands; ^8^Laboratory for Infectious Diseases and Perinatal Screening, Center for Infectious Disease Control Netherlands, National Institute for Public Health and the Environment, Bilthoven, the Netherlands; ^9^Departments of Pathology, Orthopaedic Surgery and the Spine Institute, Cleveland Clinic Foundation, Cleveland, OH, USA; ^10^Department of Orthopaedics, Kobe University Hospital, Kobe, Japan

## Abstract

**Purpose:**

We investigated the hypothesis that many total hip arthroplasty revisions that are classified as aseptic are in fact low-grade infections missed with routine diagnostics.

**Methods:**

In 7 Dutch hospitals, 176 consecutive patients with the preoperative diagnosis of aseptic loosening of their total hip arthroplasty were enrolled. During surgery, between 14 and 20 tissue samples were obtained for culture, pathology, and broad-range 16S rRNA PCR with reverse line blot hybridization. Patients were classified as either not being infected, suspected of having infection, or infected according to strict, predefined criteria. Each patient had a follow-up visit after 1 year.

**Results:**

7 patients were classified as infected, 4 of whom were not identified by routine culture. 15 additional patients were suspected of having infection. 20 of these 22 patients received a cemented prosthesis, fixated with antibiotic-loaded bone cement. All 22 patients received prophylactic systemic antibiotics. 7 of them reported complaints one year after surgery, but only one showed signs of early loosening. However, additional surgery was not performed in any of the patients.

**Interpretation:**

Although the proportions were not as high as previously reported in the literature, between 4% and 13% of patients with the preoperative diagnosis of aseptic loosening were infected. However, as thorough debridement was performed during surgery and prophylactic antibiotics were used, the diagnosis of infection did not have any obvious clinical consequences, as most patients performed well at the 1-year follow-up. Whether this observation has implications for long-term implant survival remains to be seen.

Despite improvements in multiple factors that influence the outcome of total hip arthroplasty, approximately 8% of prostheses need to be revised within 10 years. More than 75% of these revisions are due to aseptic loosening, whereas primary or secondary infection accounts for approximately 8% (Malchau et al. 2002). Data gathered by the Scandinavian hip registries clearly suggest that antibiotic prophylaxis—either systemically, locally, or combined—increases implant survival. In fact, the widespread use of antibiotic-containing bone cement in primary total hip arthroplasties in these countries has been associated with a decrease in the number revisions due to aseptic loosening as well (Malchau et al. 2002, Engesaeter et al. 2003). As aseptic loosening should not be influenced by the activity of antibiotics, this observation suggests that there is underdiagnosis of infection. The aseptic loosening group might contain a substantial number of patients with low-grade infections, which are missed with routine diagnostic tests. As a result, these patients might not get an optimal treatment, which might ultimately lead to complications. Other publications have presented evidence of bacteria or bacterial DNA in patients thought to have developed aseptic loosening (Mariani et al. 1996, Tunney et al. 1999, Neut et al. 2003, Clarke et al. 2004, Ince et al. 2004, Trampuz et al. 2006, Moojen et al. 2007). However, the prevalence of bacterial involvement in revision surgery has ranged from just a few per cent up to 70% of all patients (Tunney et al. 1999, Trampuz et al. 2006).

A difficulty with confirming the diagnosis of infection in total hip arthroplasty is that there is no gold standard (Bauer et al. 2006). Traditionally, microbiological cultures are seen as the reference standard, but these are often false positive due to contamination or false negative due to changed growth characteristics of bacteria. Obtaining too few samples or from inappropriate biopsy sites may also result in false negative results. Histopathological analysis of tissue samples is frequently used (Spangehl et al. 1999, Bauer et al. 2006). However, there is no consensus on which number of polymorphonuclear leukocytes is the best cut-off value (Mirra et al. 1982, Lonner et al. 1996, Pandey et al. 2000). The best reference would probably be the combined results of several diagnostic tests (Atkins et al. 1998, Spangehl et al. 1999, Bauer et al. 2006).

In recent years, different studies on new, more sensitive diagnostic techniques have been published. The use of molecular biological techniques in particular, such as polymerase chain reaction (PCR), has been investigated (Mariani et al. 1996, Tunney et al. 1999, Clarke et al. 2004, Fenollar et al. 2006, Kobayashi et al. 2006, Moojen et al. 2007). PCR techniques are, however, very susceptible to contamination. The key publications by Mariani et al. (1996) and Tunney et al. (1999) therefore probably also contained a substantial percentage of false positive results, as was acknowledged by the authors in a later publication (McDowell and Patrick 2005). Moojen et al. (2007) published a study on the optimization and validation of a combined broad-range 16S rRNA PCR and reverse line blot hybridization protocol. With this technique, several microorganisms often encountered in orthopedic infections could be reliably detected and identified at the genus and species level, and the combined PCR-RLB technique was found to be more sensitive than routine culture.

Our hypothesis was that there is an underdiagnosis of infection in patients undergoing revision total hip arthroplasty. We investigated this by extensive use of routine diagnostics such as microbiological culture and pathological analysis, together with the previously validated broad-range 16S rRNA PCR and reverse line blot hybridization technique in a group of patients who were suspected of having aseptic loosening preoperatively.

## Patients and methods

### Study design

This study was approved by the institutional board review committee of the University Medical Center Utrecht (13-09-2003, 03/133). Between November 2002 and July 2006, 176 patients with the preoperative diagnosis of aseptic loosening of their total hip arthroplasty were admitted to 7 Dutch hospitals. All were scheduled for a 1-stage revision of cup, stem, or both. During surgery, several tissue biopsies were obtained and sent for microbiological culture, pathological analysis, and broad-range 16S rRNA PCR with reverse line blot hybridization. Each patient had a 1-year follow-up visit.

### Eligibility for inclusion

Patients were eligible for inclusion if they were at least 18 years old and were scheduled for a revision of either one or both components of their total hip arthroplasty, because of a preoperative diagnosis of aseptic loosening. This diagnosis had to be based on at least anamnesis, physical examination, anteroposterior and lateral radiographs of the involved hip, and the determination of erythrocyte sedimentation rate (ESR) and C-reactive protein (CRP). Both first and re-revision cases were included. All patients who were enrolled had provided written informed consent. Exclusion criteria were indication for revision surgery because of technical error, recurrent dislocation, periprosthetic fracture, or solely polyethylene wear (e.g. only exchange of a liner); history of septic arthritis, osteomyelitis or deep infection of the symptomatic hip or a clinically apparent infection at another site. Finally, antibiotic use less than 2 weeks before revision surgery was also a reason for exclusion.

### Patients

176 patients (127 women) were included (Table 1). Median age at the time of revision surgery was 72 (28–92) years. The indication for primary total hip arthroplasty was osteoarthritis in 115 patients, sequelae of childhood disease in 21, fracture in 10, inflammatory arthritis in 10, osteonecrosis in 9, and other reasons or unknown in 11. In 130 patients, it was a first revision. The median time the prosthesis had been in place was 11 (0.5–35) years. Regarding fixation of the original implant, it was cemented in 83 patients, hybrid in 19, and uncemented in 74. In most cases where bone cement had been used for the components now being revised, it had contained either tobramycin or gentamicin. However, because of many referrals, the antibiotic content of this cement was unknown in about half of the cases. Prophylactic systemic antibiotics had been used during the previous surgery in 71 of 79 patients; this information was unknown for the other 97 patients. A first- or second-generation cephalosporin was used in 75%; other antibiotics included clindamycin and amoxicillin/clavulanic acid.

**Table 1.** Patient demographics, surgical history, and diagnostics

Age, mean (range)	70 (28–92)
Males/females	49/127 (28%/72%)
Indication for THA	
osteoarthritis	115 (65%)
childhood disease	21 (12%)
fracture	10 (6%)
inflammatory arthritis	10 (6%)
osteonecrosis	9 (5%)
unknown	9 (5%)
other	2 (1%)
Revision number	
1st	130 (74%)
2nd	34 (19%)
3rd	8 (5%)
4th	3 (2%)
5th	1 (1%)
Years in situ of arthroplasty, mean (range)	11 (0.5–35)
Method of fixation	
cemented	83 (47%)
hybrid	19 (11%)
uncemented	74 (42%)
Systemic antibiotics at previous surgery	
yes	71 (40%)
no	8 (5%)
unknown	97 (55%)
Anamnesis	
Pain	161 (91%)
Functional impairment	90 (51%)
Radiography	
Normal	21 (12%)
Loosening of cup	74 (42%)
Loosening of stem	47 (27%)
Loosening of both components	34 (19%)
Blood analysis	
ESR, mean (range)	17 (1–90)
CRP, mean (range)	5.1 (< 1–45)
Additional diagnostics	
No	49 (28%)
Aspiration and arthrography	53 (30%)
Scintigraphy	96 (55%)

90% of patients complained of pain, and half of them reported functional impairment. Radiographs showed signs of loosening of one or both components in 88% of patients; none showed signs suggestive of infection. Mean ESR was 17 (SD 14) and mean CRP was 5 (SD 2). In 72% of the patients, additional diagnostic tests were performed, including aspiration and arthrography in 30% and scintigraphy in 55%.

In 63 patients both components were revised; in 76 only the acetabular cup was revised, and in 37 only the femoral stem was revised. All patients received intravenous antibiotics perioperatively (a cephalosporin, clindamycin, or amoxicillin/clavulanic acid), but only after obtaining the tissue samples.

### Sample acquisition and analysis

Preoperatively, for each patient the medical history was obtained, plain radiographs were taken, and ESR and CRP levels were determined. In case of doubt, the treating orthopedic surgeon was free to perform additional diagnostics, such as aspiration and arthrography or scintigraphy, but this was not obligatory for the study. During surgery, prophylactic antibiotics were withheld until sampling was done. Depending on whether a partial or total revision was performed, between 14 and 20 samples were obtained from the joint fluid, the (neo)capsule, and the acetabular and femoral interface, resulting in 4–7 samples for each diagnostic test. Joint fluid was aspirated prior to opening the capsule. Capsule samples were taken from the articular side of the (neo)capsule, at the site that appeared most inflamed. Interface samples were taken at the sites that showed most osteolysis on the preoperative radiographs. The sampling was done according to a strict protocol and for each location clean instruments and gloves were used. To minimize tissue handling, a sterile sample kit was available for each patient. Samples for culture were analyzed at the local microbiology laboratory. In each hospital, the samples were cultured according to similar protocols, which meant culturing on solid and liquid culture media for both aerobic and anaerobic microorganisms for at least 7 days. As we aimed to mimic clinical practice, there were minor differences between the exact culture protocols of the different hospitals. Samples for pathological analysis were immersed in 37% formaldehyde and processed at the local pathology laboratory. Samples were embedded in paraffin and hematoxylin/eosin-stained slides were made. All slides were analyzed centrally by 1 study pathologist (TB), who was specialized in histopathology of failed implants and who was blinded with respect to patient identification and the results of all other tests. Criteria for infection were modified from those of Spangehl et al. (1999). When a maximum tissue concentration of 0–5 polymorphonuclear neutrophils (PMNs) per 10 high power fields (HPF; 400× magnification, field diameter 0.54 mm) was seen, this was regarded as definitely not infected. When a maximum tissue concentration of 6–10 PMNs per 10 HPFs was seen, this was regarded as borderline, but probably not infected. When > 10 PMNs per 10 HPFs, but not > 5 PMNs in any one HPF was seen, this was regarded as borderline, but most likely infected. When > 5 PMNs per HPF were seen, this was regarded as definitely infected. PMNs entrapped in superficial fibrin or in capillaries were excluded (Bauer et al. 2006).

Samples for the broad-range 16S rRNA PCR with reverse line blot hybridization (PCR-RLB) were stored locally at –70°C and analyzed centrally, according to the techniques previously validated and published by our group (Moojen et al. 2007). Briefly, the technique consists of a broad-range 16S rRNA PCR using universal primers targeted at the 16S rRNA gene, which amplifies part of the bacterial DNA. PCR products are detected and characterized further at the genus or species level using reverse line blot hybridization, which uses 28 oligonucleotide probes that were specifically designed for the detection of bacteria associated with joint infections. Only a positive result on 2 separate PCR-RLB runs was regarded as positive.

One year postoperatively, each patient had a follow-up visit where postoperative clinical course, additional surgery, or antibiotic use was recorded, radiographs were taken, and ESR and CRP were determined.

### Diagnosis of infection

Patients were regarded as infected, suspected of being infected, or as not being infected. A patient was regarded as infected if he or she met the criteria as described by either Spangehl et al. (1999) or Atkins et al. (1998). According to Spangehl, a patient is infected when there is either an open wound or sinus to the joint, a systemic infection with pain in the hip and purulent fluid in the joint, or a positive result for at least 3 diagnostic tests for infection: a CRP of > 10, an ESR of > 30, a positive preoperative aspiration, ≥ 5 PMNs per HPF on pathological analysis, or at least one third of the culture samples positive for the same microorganism. The PCR-RLB was added to these tests, using the same criteria as for culture. Atkins investigated the value of microbiological results as a single diagnostic approach. According to these criteria, at least 3 samples must be positive for the same microorganism to be regarded as being truly infected. For the PCR-RLB results, this definition was used in the same manner. As we were searching for low-grade infections, a patient was considered suspect regarding infection if he or she had either 2 culture results or 2 PCR-RLB results positive for the same microorganism, or had a pathological analysis that definitely showed infection. All other patients were regarded as not being infected.

## Results

### Uninfected patients

In 127 patients (72%), every sample obtained during surgery was negative by culture (Table 2). Similarly, none of the samples were positive by PCR-RLB in 141 patients (80%). Pathological analysis suggested that there was no infection in 157 patients (89%). If the 3 tests were combined, they were all negative in 98 patients (56%). Although many patients had a positive result in 1 of the 3 tests (Table 2), not all of the remaining 78 patients were considered infected. In many patients, only 1 or 2 samples were positive by culture or PCR-RLB, or different microorganisms were cultured from different samples. Thus, these results were regarded as being due to contamination and were interpreted as being false positive. With respect to cultures, 46 patients (26%) were regarded as being false positive, 28 patients (16%) in the PCR-RLB and 14 patients (8%) in the pathological analysis. When the results of the 3 tests were combined, 71 patients (40%) had a false positive result in 1 or more diagnostic tests. According to the criteria used, 154 of the 176 patients (88%) were classified as not being infected.

**Table 2.** Results of diagnostic tests on samples from 176 patients

	All samples negative		One or more samples positive		True positives	
	n	%	n	%	n	%
Microbiology	127	72	49	28	3	2
PCR-RLB	141	80	35	20	7	4
Pathology	157	89	19	11	5	3
Combined	98	56	78	44	7	4

### Infected patients

Culture was regarded as true positive in 3 patients (2%), PCR-RLB in 7 patients (4%), and pathological analysis in 5 patients (3%). According to the strict criteria used for infection, 7 patients (4%) were classified as infected (Table 3). 6 of these 7 patients had positive results in several diagnostic analyses, and were classified as infected according to the criteria of Spangehl. Only 1 additional patient was classified as infected according to the criteria of Atkins. In this patient, 3 of his 5 PCR-RLB samples were positive for Enterobacter agglomerans, while other tests were negative. Coagulase-negative staphylococci were found in 3 patients and Proprionibacterium acnes, Streptococcus spp., Salmonella spp., and E. agglomerans were found in 1 patient each. With culture, a microorganism was found in only 3 of the 7 infected patients whereas all were detected with PCR-RLB. The positive PCR-RLB results were supported in all but 1 case, with pathology results giving rise to suspicion of infection.

**Table 3.** Results of diagnostic tests on samples from patients who were considered to be infected

Patient	ESR	CRP	Aspiration	Culture	Pathology	PCR-RLB ^a^
1	55	18	not done	Gram+ cocci (2 of 5)	negative	*Staphylococcus sp*./CNS (1 of 6)
2	35	10	not done	negative	positive	*Salmonella sp.* (3 of 4)
3	17	< 5	not done	negative	negative	*E. agglomerans* (3 of 5)
4	34	< 5	not done	negative	positive	*P. acnes* (4 of 4)
5	20	4	not done	*S. epidermidis* (3 of 5)	positive	*Staphylococcus sp*./CNS (4 of 4)
6	12	11	negative	Skin flora (5 of 7)	positive	*Staphylococcus sp*./CNS (5 of 6)
7	31	21	not done	negative	positive	*Streptococcus sp*. (3 of 7)

**^a^** CNS: coagulase-negative staphylococci

### Patients suspected of being infected

As the types of infections searched for in the study group would most likely be low-grade, it is possible that the previously described criteria for infection were too strict. Less strict criteria identified 15 additional patients with suspected infection (Table 4); 8 cases were based on culture, 2 on PCR-RLB, 1 on both culture and PCR-RLB, and 4 on pathology analysis. Microorganisms found in these patients were *P. acnes* in 4, coagulase-negative staphylococci in 2, *Streptococcus spp.* in 2, and *Staphylococcus aureus*, *Micrococcus spp.,* or a diphteroid rod in the other 3 patients.

**Table 4.** Results of diagnostic tests on samples from patients who were suspected of being infected

Patient	ESR	CRP	Aspiration	Culture	Pathology	PCR-RLB ^a^
8	11	1	not done	negative	negative	*Staphylococcus sp*./CNS (2 of 7)
9	11	5	not done	*Micrococcus sp*. (2 of 6)	negative	negative
10	5	2	not done	*S. aureus* (2 of 7)	negative	negative
11	10	6	not done	*Str. kominis* (2 of 5)	negative	negative
12	13	2	not done	*P. acnes* (2 of 4)	negative	negative
13	25	2	not done	*P. acnes* (2 of 5)	negative	negative
14	9	9	negative	Difteroid rod (2 of 5)	negative	negative
15	14	< 5	not done	*P. acnes* (2 of 5)	negative	negative
16	3	< 5	not done	CNS (2 of 7)	negative	*Staphylococcus sp*./CNS (2 of 7)
17	13	13	negative	negative	negative	*P. acnes* (2 of 6)
18	8	< 7	negative	Negative	positive	negative
19	10	< 7	negative	*Streptococcus sp.* and *Corynebacterium sp.* (both 2 of 4)	negative	negative
20	7	6	not done	negative	positive	negative
21	7	10	not done	negative	positive	negative
22	23	4	not done	negative	positive	negative

**^a^** CNS: coagulase-negative staphylococci

### Postoperative clinical course and 1-year follow-up

Of the initial group of 176 patients, information from 1-year follow-up was available for 170 (97%). As none of the patients were suspected of having infection preoperatively, all were scheduled for a 1-stage revision and for only a short course of prophylactic systemic antibiotics (range 1–5 days). As the PCR-RLB and pathology results were not available to the treating orthopedic surgeon, these did not influence decisions for postoperative treatment. Because of strong intraoperative suspicion of infection, a 2-stage revision was performed in 3 patients, only 1 of whom was indeed infected.

#### Uninfected patients

In the 148 patients that were not suspected of having an infection, 146 had a 1-stage revision. 5 patients received prolonged treatment with antibiotics because of positive intraoperative cultures or persistent postoperative wound drainage. 9 patients had additional surgery after the 1-stage revision. In 4 patients, a debridement was performed because of suspected infection. 4 patients had a 1-stage revision because of technical problems caused by the previous operation, and 1 had a 2-stage re-revision because the clinical course was complicated by infection. At the 1-year follow-up, 121 patients had no complaints. 27 patients did have complaints, 21 of whom showed no signs of infection or loosening, 5 showed signs of implant loosening, and 1 had a resection arthroplasty.

#### Infected patients

In the 7 patients who were infected, all but 1 had a 1-stage revision. 6 patients received an implant fixated with antibiotic bone cement and 1 patient received an uncemented implant. Only the patient with the 2-stage revision received antibiotics for a prolonged period. After 1 year, only 2 patients reported complaints, but there were no signs of loosening or infection. No debridement or re-revision was performed.

#### Patients suspected of being infected

The 15 additional patients who were suspected of having an infection using less strict criteria all had a 1-stage revision. 14 patients received an implant fixated with antibiotic bone cement and 1 patient received an uncemented implant. 1 patient received prolonged antibiotics because of positive intraoperative cultures, and did well at follow-up. After 1 year, 4 patients reported complaints without signs of infection or loosening. 1 patient showed loosening of the cup with an elevated ESR and CRP, and IgG scintigraphy suggestive of low-grade infection. However, no additional surgery was performed in this group either.

## Discussion

This prospective study of 176 patients who underwent revision surgery of their total hip arthroplasty because of the preoperative diagnosis of aseptic loosening, showed that in 7–22 patients (4–13%) there was infection or suspicion of infection, based on the combined results of pre- and intraoperative diagnostic tests for infection. All patients but one had a 1-stage revision and they were treated accordingly; only 2 patients received antibiotics over a prolonged period. At the 1-year follow-up, most patients did well. None of these 22 patients had received additional surgery after 1 year.

Compared to several previous studies that have investigated a similar hypothesis, we found a relatively low rate of infection. For example, in a similar group of patients Tunney et al. (1999) showed that there were bacteria in 22% using sonication and culture, in 63% using immunofluorescence microscopy, and in 72% using 16S PCR. Using 16S PCR, Clarke et al. (2004) reported evidence of bacterial DNA in half of their patients with suspected aseptic loosening. However, they found bacterial DNA in 21% of the tissue samples from primary total hip arthroplasties as well, indicating a high contamination rate. Mariani et al. (1996) found similar results in a group of 50 patients with aseptic and septic revision of total knee implants. In addition to the 15 patients identified by culture, 16S PCR revealed the presence of bacterial DNA in 17 additional patients. As their negative controls were negative, they considered the 17 patients to be true positives. The problem with these studies is that neither of them used a valid reference standard for infection, but only reported percentages of patients who had positive samples. In contrast, the 2 large studies previously mentioned regarding definition of a reference standard for infection did not concentrate on the identification of low-grade infections (Atkins et al. 1998, Spangehl et al. 1999).

Although we tried to use the best published reference standards for infection, this will always be a topic of debate. Most studies have been designed to detect standard infections with good sensitivity and specificity, and have used internal references for comparison. For example, Atkins et al. (1998) used the pathology diagnosis as the gold standard to which they compared the culture results. In our study, we anticipated low-grade infections, which would probably be difficult to detect. Using published criteria, we identified only 7 infected patients (4%), which suggests that these criteria have good specificity. Lowering of the stringency for diagnosis of infection in an attempt to identify more low-grade infections would be expected to increase the sensitivity, but decrease the specificity. In our study, it increased the number of patients suspected of being infected to 22 (13%). Perhaps the identification of the same microorganism in 2 separate tissue samples or only a high concentration of polymorphonuclear neutrophils in peri-implant tissues would increase the sensitivity. On the other hand, it is possible that 4% might just be the true incidence.

The types of microorganisms found in the infected patients consisted mainly of low-virulence bacteria such as coagulase-negative staphylococci and Proprionibacterium acnes. These bacteria are skin flora, but they are also often associated with orthopedic implant-related infections (Zimmerli et al. 2004, Fenollar et al. 2006, Zappe et al. 2008). In one infected patient, a Salmonella species was found in 3 PCR-RLB samples. Salmonella is a microorganism not often related to infections of total hip arthroplasties, but it has been reported previously (Chong and Sporer 2005). In some patients conventional cultures were negative, yet the PCR-RLB yielded positive results. The validity of the PCR-RLB results was supported by the pathology results, which indicated infection as well. Regarding the detection of microorganisms, there was a distinct difference between culture and PCR-RLB. PCR-RLB detected the presence of bacterial DNA in all 7 infected patients, even though only 1 of 6 samples from 1 patient was positive for coagulase-negative staphylococci. In contrast, routine cultures identified a microorganism in only 3 of the 7 patients and could only specify the type of microorganism found as Gram-positive cocci and skin flora in 2 patients. PCR-RLB showed coagulase-negative staphylococci in both. This low sensitivity of routine cultures may be related to a more fastidious growth of these bacteria. However, it must be said that by using new sonication techniques and prolonged culture periods, these fastidious microorganisms can be detected more reliably and the sensitivity of culture is improved (Neut et al. 2003, Trampuz et al. 2007).

Although polymicrobial infections have been reported to occur frequently in implant-related infections, we found only one patient suspected of having an infection in whom more than 1 type of microorganism was identified. In this patient, routine culture identified both Streptococcus species and Corynebacterium species, while all PCR-RLB samples were negative. Even though we detected no polymicrobial infections with PCR-RLB, the combined technique has also been found to identify this type of infection reliably (Moojen et al. 2007). With the reverse line blot hybridization technique, oligonucleotide probes corresponding to different microorganisms are bound to the detection membrane; thus, several bacterial PCR products can be identified in one assay.

One point of criticism might be that a preoperative aspiration was performed in only a limited number of patients (30%), which does correpond to normal clinical practice in many hospitals. As it is an invasive procedure, this was not obligatory for the study and was only performed by the treating orthopedic surgeon in cases of doubt. An aspiration could have added valuable information, which could in turn have changed treatment strategies. On the other hand, in all patients in whom aspirations were performed, the results were negative.

Even though we found that a substantial proportion of patients were infected or were suspected of being infected in a group that was not thought to be infected preoperatively, the consequences of this finding remain to be seen. Almost all patients were treated with a 1-stage revision and only one to a few days of prophylactic systemic antibiotics, but at the 1-year follow-up none of those 22 patients had received any additional surgery to the revised hip. Compared to the group of patients who were not suspected of being infected, they fared similarly. One patient who was suspected of having an infection did have complaints and showed signs suggestive of low-grade infection at one year. A 1-stage revision with thorough intraoperative debridement and the prophylactic use of systemic and perhaps additional local antibiotics in the bone cement might be sufficient treatment for most low-grade infections with low-virulence bacteria. Although most of our patients received a cemented revision prosthesis fixated with antibiotic-loaded bone cement, the patients with an uncemented prosthesis did not appear to do worse, so no hard and fast statements can be made regarding the potential additional value of local antibiotics. However, even though using antibiotic-loaded bone cement has proven valuable for infection prophylaxis and implant survival (Engesaeter et al. 2003), it should always be remembered that by the routine use of this type of cement, bacterial resistance to antibiotics such as gentamicin and tobramycin can be induced.

In summary, we that found in 176 patients who required revision surgery of their total hip arthroplasty and who were not suspected of having infection preoperatively, between 4% and 13% came to be regarded as infected, based on the combined results of culture, histology, and broad-range 16S rRNA PCR with reverse line blot hybridization diagnostics. These infections were mainly caused by low-virulence bacteria, for which detection with PCR-RLB was more sensitive than routine culture. For implant survival after 1 year, the presence of infection seemed to be of little consequence. However, 1 year is still too short a follow-up interval for us to be fully confident about the absence of low-grade infection, so the consequences for long-term survival remain to be seen.

## References

[CIT0001] Atkins BL, Athanasou N, Deeks JJ, Crook DW, Simpson H, Peto TE, McLardy-Smith P, Berendt AR (1998). Prospective evaluation of criteria for microbiological diagnosis of prosthetic-joint infection at revision arthroplasty. The OSIRIS Collaborative Study Group. J Clin Microbiol.

[CIT0002] Bauer TW, Parvizi J, Kobayashi N, Krebs V (2006). Diagnosis of periprosthetic infection. J Bone Joint Surg (Am).

[CIT0003] Chong PY, Sporer SM (2005). Case report: Salmonella infection following total hip arthroplasty. Iowa Orthop J.

[CIT0004] Clarke MT, Roberts CP, Lee PT, Gray J, Keene GS, Rushton N (2004). Polymerase chain reaction can detect bacterial DNA in aseptically loose total hip arthroplasties. Clin Orthop.

[CIT0005] Engesaeter LB, Lie SA, Espehaug B, Furnes O, Vollset SE, Havelin LI (2003). Antibiotic prophylaxis in total hip arthroplasty: effects of antibiotic prophylaxis systemically and in bone cement on the revision rate of 22,170 primary hip replacements followed 0-14 years in the Norwegian Arthroplasty Register. Acta Orthop Scand.

[CIT0006] Fenollar F, Roux V, Stein A, Drancourt M, Raoult D (2006). Analysis of 525 Samples To Determine the Usefulness of PCR Amplification and Sequencing of the 16S rRNA Gene for Diagnosis of Bone and Joint Infections. J Clin Microbiol.

[CIT0007] Ince A, Rupp J, Frommelt L, Katzer A, Gille J, Lohr JF (2004). Is “aseptic” loosening of the prosthetic cup after total hip replacement due to nonculturable bacterial pathogens in patients with low-grade infection?. Clin Infect Dis.

[CIT0008] Kobayashi N, Bauer TW, Tuohy MJ, Lieberman IH, Krebs V, Togawa D, Fujishiro T, Procop GW (2006). The comparison of pyrosequencing molecular Gram stain, culture, and conventional Gram stain for diagnosing orthopaedic infections. J Orthop Res.

[CIT0009] Lonner JH, Desai P, Dicesare PE, Steiner G, Zuckerman JD (1996). The reliability of analysis of intraoperative frozen sections for identifying active infection during revision hip or knee arthroplasty. J Bone Joint Surg (Am).

[CIT0010] Malchau H, Herberts P, Eisler T, Garellick G, Soderman P (2002). The Swedish Total Hip Replacement Register. J Bone Joint Surg (Am) (Suppl 2).

[CIT0011] Mariani BD, Martin DS, Levine MJ, Booth RE, Tuan RS (1996). The Coventry Award. Polymerase chain reaction detection of bacterial infection in total knee arthroplasty. Clin Orthop.

[CIT0012] McDowell A, Patrick S (2005). Evaluation of nonculture methods for the detection of prosthetic hip biofilms. Clin Orthop.

[CIT0013] Mirra JM, Marder RA, Amstutz HC (1982). The pathology of failed total joint arthroplasty. Clin Orthop.

[CIT0014] Moojen DJ, Spijkers SN, Schot CS, Nijhof MW, Vogely HC, Fleer A, Verbout AJ, Castelein RM, Dhert WJ, Schouls LM (2007). Identification of orthopaedic infections using broad-range polymerase chain reaction and reverse line blot hybridization. J Bone Joint Surg (Am).

[CIT0015] Neut D, van Horn JR, van Kooten TG, van der Mei HC, Busscher HJ (2003). Detection of biomaterial-associated infections in orthopaedic joint implants. Clin Orthop.

[CIT0016] Pandey R, Berendt AR, Athanasou NA (2000). Histological and microbiological findings in non-infected and infected revision arthroplasty tissues. The OSIRIS Collaborative Study Group. Oxford Skeletal Infection Research and Intervention Service. Arch Orthop Trauma Surg.

[CIT0017] Spangehl MJ, Masri BA, O'Connell JX, Duncan CP (1999). Prospective analysis of preoperative and intraoperative investigations for the diagnosis of infection at the sites of two hundred and two revision total hip arthroplasties. J Bone Joint Surg (Am).

[CIT0018] Trampuz A, Piper KE, Hanssen AD, Osmon DR, Cockerill FR, Steckelberg JM, Patel R (2006). Sonication of explanted prosthetic components in bags for diagnosis of prosthetic joint infection is associated with risk of contamination. J Clin Microbiol.

[CIT0019] Trampuz A, Piper KE, Jacobson MJ, Hanssen AD, Unni KK, Osmon DR, Mandrekar JN, Cockerill FR, Steckelberg JM, Greenleaf JF, Patel R (2007). Sonication of removed hip and knee prostheses for diagnosis of infection. N Engl J Med.

[CIT0020] Tunney MM, Patrick S, Curran MD, Ramage G, Hanna D, Nixon JR, Gorman SP, Davis RI, Anderson N (1999). Detection of prosthetic hip infection at revision arthroplasty by immunofluorescence microscopy and PCR amplification of the bacterial 16S rRNA gene. J Clin Microbiol.

[CIT0021] Zappe B, Graf S, Ochsner PE, Zimmerli W, Sendi P (2008). Propionibacterium spp. in prosthetic joint infections: a diagnostic challenge. Arch Orthop Trauma Surg.

[CIT0022] Zimmerli W, Trampuz A, Ochsner PE (2004). Prosthetic-joint infections. N Engl J Med.

